# Parental high dietary arachidonic acid levels modulated the hepatic transcriptome of adult zebrafish (*Danio rerio*) progeny

**DOI:** 10.1371/journal.pone.0201278

**Published:** 2018-08-02

**Authors:** Anne-Catrin Adam, Kaja Helvik Skjærven, Paul Whatmore, Mari Moren, Kai Kristoffer Lie

**Affiliations:** Institute of Marine Research, Nordnes, Bergen, Norway; University of Illinois, UNITED STATES

## Abstract

Disproportionate high intake of n-6 polyunsaturated fatty acids (PUFAs) in the diet is considered as a major human health concern. The present study examines changes in the hepatic gene expression pattern of adult male zebrafish progeny associated with high levels of the n-6 PUFA arachidonic acid (ARA) in the parental diet. The parental generation (F_0_) was fed a diet which was either low (control) or high in ARA (high ARA). Progenies of both groups (F_1_) were given the control diet. No differences in body weight were found between the diet groups within adult stages of either F_0_ or F_1_ generation. Few differentially expressed genes were observed between the two dietary groups in the F_0_ in contrast to the F_1_ generation. Several links were found between the previous metabolic analysis of the parental fish and the gene expression analysis in their adult progeny. Main gene expression differences in the progeny were observed related to lipid and retinoid metabolism by PPARα/RXRα playing a central role in mediating changes to lipid and long-chain fatty acid metabolism. The enrichment of genes involved in *β-*oxidation observed in the progeny, corresponded to the increase in peroxisomal *β*-oxidative degradation of long-chain fatty acids in the parental fish metabolomics data. Similar links between the F_0_ and F_1_ generation were identified for the methionine cycle and transsulfuration pathway in the high ARA group. In addition, estrogen signalling was found to be affected by parental high dietary ARA levels, where gene expression was opposite directed in F_1_ compared to F_0_. This study shows that the dietary n-3/n-6 PUFA ratio can alter gene expression patterns in the adult progeny. Whether the effect is mediated by permanent epigenetic mechanisms regulating gene expression in developing gametes needs to be further investigated.

## Introduction

In today’s dietary pattern, we observe a selective decrease of n-3 polyunsaturated fatty acids (PUFAs) in favour of n-6 PUFAs. This results in a decreased n-3/n-6 PUFA ratio [1, 2]. Physiological effects of a decreasing n-3/n-6 PUFA ratio are diverse, but taken together, studies indicate that a disproportional high intake of n-6 PUFAs may contribute to health problems [3–7]. In the past decades, several studies have shown the benefit of increased n-3 PUFA levels in the diet [8, 9], and focus has been directed on n-3 PUFAs such as eicosapentaenoic acid (EPA, 20:5n-3) and docosahexaenoic acid (DHA, 22:6n-3). Arachidonic acid (ARA, 20:4n-6), an n-6 PUFA that competes for the same enzymes and therefore is interlinked, has been less studied. ARA and its derivatives, have vital roles in growth and various signalling cascades regulating inflammatory processes, bone metabolism and reproduction as observed in different species [10–14]. n-3 and n-6 PUFAs have the potential to change cellular phenotypes by changing membrane lipid composition and controlling gene expression through activating nuclear receptors [15–17]. In addition, n-3 and n-6 PUFAs can also affect DNA methylation patterns [18–20]. Recently, we demonstrated that high dietary ARA levels fed to zebrafish affected the levels of oxidized amino acids and lipids, and changed the immune-related eicosanoids and lipid metabolism [21]. More and more studies indicate that diet also affect following generations in terms of long-term health of the progeny [22–26]. Here, we investigate whether high ARA given to the parents’ generation can impact the progeny’s transcriptome. However, little is known about how changes in the parental dietary n-3 and n-6 PUFA composition impact the adult progeny.

The period of oocyte and spermatozoa maturation displays a sensitive window, where parental nutrition has metabolic influence on future fertilized eggs [27]. Another way that parental diet effects can be mediated is through transcripts deposited in the newly fertilized egg that regulate early embryonic development and thus determine future gene expression patterns, growth and physiology [28–31]. It has been shown in zebrafish that dietary micronutrient status of the parents can influence gene expression patterns of their embryos and livers of their adult offspring [32–34]. Furthermore, nutritional induced obesity of the parents has shown to affect fertility (egg production) and gene expression of zebrafish eggs [35]. Studies on different vertebrate species have demonstrated that the maternal dietary n-3 and n-6 PUFA profile influenced oocyte composition, embryonic development and health of progeny [9, 36–39]. In teleost, dietary n-3 and n-6 PUFA composition was found to affect oocytes and reproductive performance [40–44], but little is known about the changes in gene expression profiles in adult progeny.

Zebrafish (*Danio rerio*), a tropical freshwater teleost fish, is an acknowledged vertebrate model organism. It has been widely used in research to increase our understanding of gene function and the importance of nutrition in outcomes related to development, health and disease in vertebrates [45–52]. In the present study, we fed parental zebrafish either a control diet (low in ARA) or a diet high in ARA, whereas progeny from both dietary groups were fed the control diet until adulthood. We wanted to investigate the impact of parental high dietary ARA levels on transcriptomic patterns in adult progeny.

## Materials and methods

### Ethical considerations

This zebrafish feeding trial was conducted in accordance with the Norwegian Animal Research Authority and approved by the Norwegian Food Safety Authority (division no. 54, reference 2012/145126) according to the current Regulation on Animal Experimentation (FOR 1996-01-15 no. 23).

### Feeding trial and zebrafish husbandry

**Fig 1:**
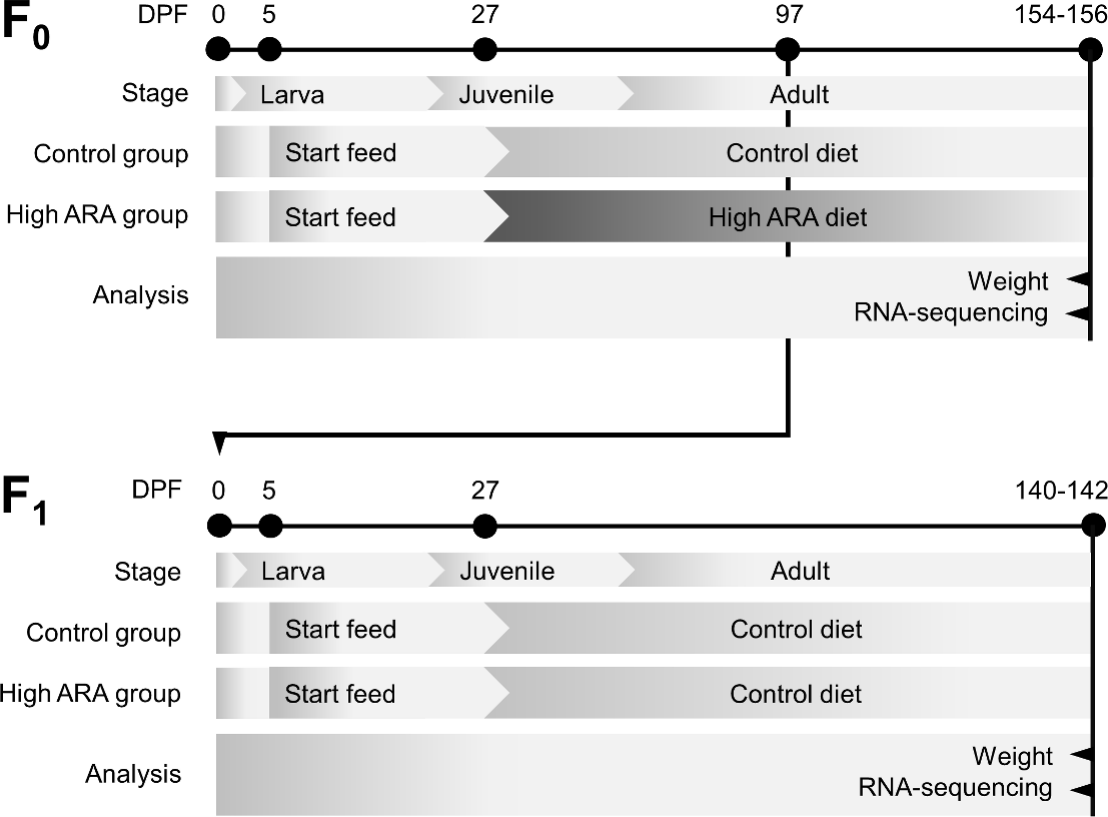
Experimental design of the transgenerational zebrafish feeding trial. F_0_ zebrafish from both control and high ARA group were fed a start feed containing Gemma micro^®^ and *Artemia* nauplii from 5 DPF until 26 DPF. The two experimental groups were given either a control or high ARA diet from 27 DPF onwards until sampling. F_0_ fish were mated at 97 DPF to produce F_1_ generation. Both groups in the F_1_ generation were fed the control diet from 27 DPF until sampling. F_0_ and F_1_ body weight (grams) and liver tissue sampling for transcriptome analysis (RNA-sequencing) were performed at 154–156 DPF (F_0_) and 140–142 DPF (F_1_).

Standardized operating procedures for mating, handling and feeding for both F_0_ and F_1_ generation of wildtype AB zebrafish (*Danio rerio*) has previously been reported [32]. Briefly, F_0_ embryos were collected randomly and larvae were fed with Gemma micro^®^ (Skretting, Stavanger, Norway) as a start feed from 5 days post fertilization (DPF) and *Artemia* nauplii (Silver Star *Artemia*, Salt Lake, USA) from 7 DPF until 26 DPF (Fig 1). The experimental diets were given twice a day from 27 DPF onwards. Control and high ARA diet composition can be found in S1 File [21]. Progeny (F_1_ generation), from both parental diet groups, were fed as the F_0_ control fish with the experimental control diet from 27 DPF until sampling. Fish were kept in 10 gender mixed tanks (containing 60 fish each until 44 DPF and thereafter reduced to 20 fish each) per diet group. All fish were kept under steadily monitored standard conditions with 28±1°C, 14 h light-10 h dark period, conductivity of 500 μS, 6 ppm (mg/L) dissolved oxygen and pH 7.5 in tanks in a reverse osmosis water treatment system (Aquatic Habitats^®^ recirculation system, MBKI Ltd, Calverton, GBR). F_0_ generation was mated at 97 DPF.

### Liver sampling and RNA extraction

Prior to dissection, fish were deprived of food for 18 h, anesthetized with 0.05% Tricaine Methane Sulphonate (PHARMAQ AS, Oslo, Norway), blotted dry on tissue paper prior to weighing, euthanized by cutting the cardinal vein and the liver was dissected subsequently. Livers were sampled in random order between 154–156 DPF (F_0_) and 140–142 DPF (F_1_) due to simultaneous sampling for other analyses connected to this trial. Six biological replicates representing six different tanks for each of the dietary groups, of which each replicate is a pool of six male livers from one tank. Livers were briefly rinsed in 1x PBS, snap frozen with liquid nitrogen and stored at -80°C for transcriptome analysis (RNA-sequencing). Total RNA was extracted using QIAzol Lysis Reagent (Qiagen, Hilden, Germany) and RNA samples were DNase treated with the Ambion^TM^ DNA-*free*^TM^ DNA Removal Kit (Invitrogen, Thermo Fisher Scientific, Waltham, USA) in order to avoid remaining genomic DNA. RNA quantity was verified using NanoDrop^®^ ND-1000 Spectrophotometer (NanoDrop Technologies, Wilmington, USA). RNA integrity (RIN) was determined using an Agilent 2100 Bioanalyser (RNA 6000 Nano LabChip kit, Agilent Technologies, Santa Clara, USA). RIN values were on average 9.06±0.39.

### RNA high-throughput sequencing and data processing

The Norwegian Sequencing Centre (NSC) performed RNA-sequencing (RNA-seq) and library preparation using TruSeq^TM^ Stranded mRNA Library Prep Kit (Illumina, Inc, San Diego, USA). Libraries were sequenced on the NextSeq500 platform (Illumina, Inc, San Diego, USA) to generate single-end 75bp reads. Sequence quality was assessed using FastQC v0.11.5. Finding high quality (Phred scores almost universally above 30) and close to zero adapter contamination on the raw reads, we decided that mapping untrimmed reads to the genome was the optimal strategy, instead allowing the mapping software to exclude errors through discarded mismatches [53]. An average of 10 047 201 reads per sample were mapped to the GRCz10 (Genome Reference Consortium Zebrafish Build 10) assembly based on both RefSeq (GCF 000002035.5 GRCz10) and Ensembl [54] using the default parameters of HISAT2 [55] resulting in an average of 76.29% of reads unambiguously assigned to RefSeq genes and 82.22% of reads unambiguously assigned to Ensembl genes. Read counts per gene were quantified using featureCounts [56] and pre-filtered to exclude combined mean read counts smaller than 10.

### Bioinformatic analysis

Differential gene expression was estimated using DESeq2 [57]. By default, internal normalization was performed to correct for variable sequencing depth and library size. Wald-test was used for significance testing and Benjamini-Hochberg for p-value false discovery correction (adjusted p). DESeq2 analysis and visualisation of data were performed in R (http://cran.rproject.org/). Mapping against different reference genomes can produce variable expression values and differentially expressed genes (DEGs) identified [58]. The annotated DEG lists from both RefSeq and Ensembl reference genomes are given in S1–S8 Tables. However, enrichment and downstream analyses were based on concordant DEGs between both reference genome annotations (S9–S12 Tables). The data discussed in this publication have been deposited in NCBI's Gene Expression Omnibus [59] and are accessible through GEO Series accession number GSE104692 (https://www.ncbi.nlm.nih.gov/geo/query/acc.cgi?acc=GSE104692).

Concordant gene symbols of DEGs (adjusted p-value<0.05) of F_1_ generation were sent to Ingenuity^®^ Pathway Analysis software suite (IPA^®^, Ingenuity Systems, Qiagen, Redwood City, USA) for downstream analysis. DEGs with corresponding adjusted p-value and log2 fold change (log2FC) were imported into IPA^®^ as human orthologues (S13 Table). An overlap p-value (right-tailed Fisher`s Exact test, p<0.05) and an activation z-score for the correlation between the imported RNA-seq dataset and the Ingenuity^®^ Knowledge Base is calculated. IPA^®^ integrates direct DEG changes to predict an upregulation or downregulation of canonical pathways and biological functions in F_1_ high ARA livers using z-scores. DEGs from the comparison of both control groups and both high ARA groups between the generations were used for functional annotation for KEGG pathways and GO terms by over-representation testing using the R package ‘clusterProfiler’ [60] (S14 Table).

### Validation of RNA-sequencing by RT-qPCR

Reverse transcription followed by quantitative real-time PCR (RT-qPCR) was performed as previously described [61] for validating the RNA-seq data. Reverse transcription and PCR of standard curve and individual samples was run with the Gene Amp 9700 PCR machine (Applied Biosystems, Foster City, USA). Real-time RT-qPCR was performed starting with a 5 min template incubation and denaturation step at 95°C, followed by 45 cycles divided in 10 s denaturation at 95°C, 10 s annealing at 60°C and 10 s synthesis at 72°C using the CFX384 Touch™ Real-Time PCR Detection System (Bio-Rad Laboratories, Inc., Hercules, USA) with the LightCycler^®^ 480 SYBR Green I Master kit (Roche Applied Science, Penzberg, Germany). Samples were amplified in triplicates and the mean was used for further calculations. Normalised expression of target genes was determined using the geNorm algorithm [62] based on the geometric mean of 3 reference genes: *eef1a1I1* (Eukaryotic translation elongation factor 1 alpha 1, like 1) [34], *tuba1c* (Tubulin, alpha 1c) [63] and *actb1* (Actin, beta 1) (S2 File). We investigated gene transcription for *fasn* (Fatty acid synthase), *mat1a* (Methionine adenosyltransferase I, alpha), *cbsb* (Cystathionine-beta-synthase b) and *vtg5* (Vitellogenin 5) that were selected among the differentially expressed genes in F_1_ livers from the RNA-seq analysis.

### Statistical analysis

Differences in body weight between the dietary groups are presented as mean with standard deviation (SD) and an unpaired, two-tailed *t-*test was used for significance testing (p-value<0.05). For validation of the RNA-seq results by real-time RT-qPCR, gene expression was tested for group differences using an unpaired, two-tailed, non-parametric *t*-test (S2 File). Statistical significance analysis of F_0_ and F_1_ body mass and RT-qPCR gene expression results was performed with GraphPad Prism 6 software (GraphPad Software, Inc, San Diego, USA).

## Results

### Body weight of F_0_ and F_1_ zebrafish

We observed no changes in body weight between the diet groups in both F_0_ and F_1_ generation (Table 1).

**Table 1:** Body weight of F_0_ zebrafish and their progeny (F_1_).

	Control (g)			High ARA (g)			
	Mean	SD	n ^1^	Mean	SD	n^1^	p ^2^
F_0_ fish (154–156 DPF)	0.44	0.05	36	0.42	0.06	36	0.19
F_1_ fish (140–142 DPF)	0.35	0.04	36	0.34	0.06	35	0.40

Fish age is given in days post fertilization (DPF).

^1^ n are individual fish originated from six tanks.

^2^ An unpaired, two-tailed *t*-test (GraphPad) was used for significance testing (p<0.05).

### Liver gene expression patterns

**Fig 2:**
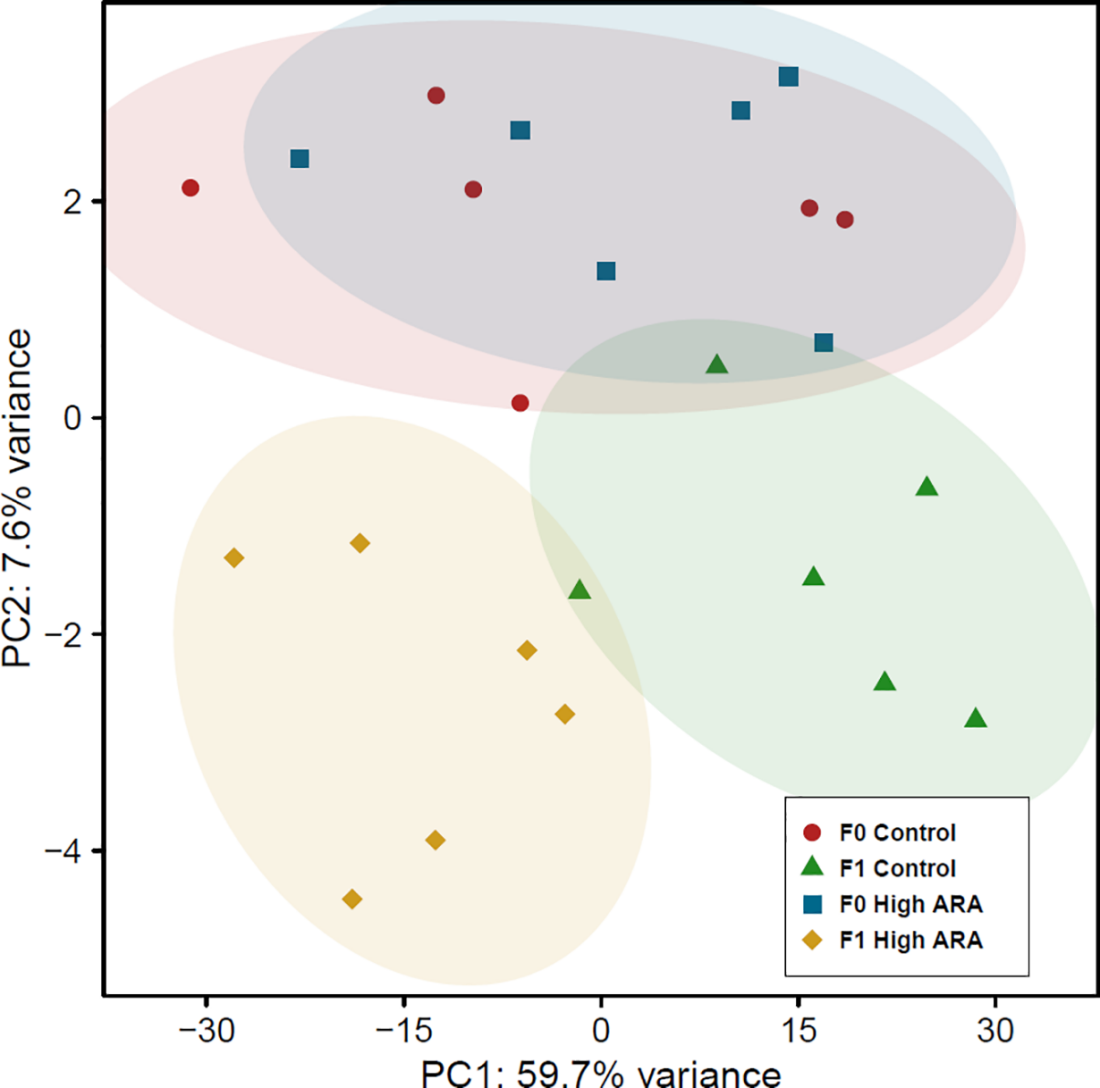
Principal component analysis of RNA-sequencing data from male livers in F_0_ generation fed either a control or high ARA diet and their F_1_ progeny fed the control diet. The plot displays high similarity in the transcriptome of F_0_ control and high ARA livers compared to clearer separation in gene expression patterns between the F_1_ diet groups. The magnitude of variation between replicates within a diet group were similar among all groups. Plot shows data underlying log-transformed read counts based on RefSeq reference genome mapping.

**Fig 3:**
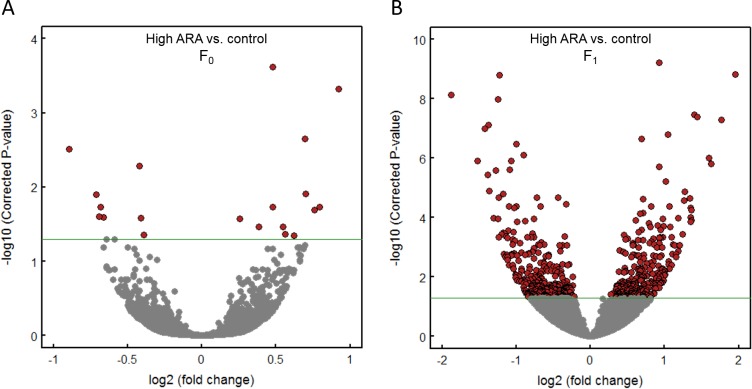
Volcano plot of RNA-sequencing data from male livers in F_0_ generation fed either a control or high ARA diet and their F_1_ progeny where both groups received the control diet. Presented data represents overlapping genes from both RefSeq and Ensembl reference genome mapping (GRCz10). Red spots represent differentially expressed genes (DEGs) between control and high ARA group in F_0_ (**A**) and F_1_ (**B**) generation. The green line denoted the significance threshold (adjusted p<0.05) for DEGs.

Principal component analysis (Fig 2) and volcano plot comparison (Fig 3) showed a clear separation between the dietary groups in F_1_. This was not as clear for the F_0_ generation. It also shows a clear separation based on generation (F_0_ vs F_1_), even between the control groups.

We found 20 DEGs between control and high ARA (adjusted p<0.05) in F_0_ generation (Fig 3A and S3 File) and 470 DEGs in F_1_ generation (Fig 3B and S3 File). Comparing F_0_ and F_1_ generation, 428 and 1987 DEGs were found to be differentially expressed between F_0_ and F_1_ control (S3 and S4 File) and between F_0_ and F_1_ high ARA groups (S3 and S4 Files), respectively.

#### ARA induced modulation of the parental (F_0_) liver transcriptome

No functional enrichment analysis was performed due to low DEG count in F_0_ generation. However, among the 20 DEGs (S9 Table), we found two affected genes relevant for lipid metabolism: *ncoa3* (nuclear receptor coactivator 3) involved in co-activation of different nuclear receptors like retinoid x receptors (RXRs) and peroxisome proliferator-activated receptors (PPARs), and *bbox1* (gamma-butyrobetaine hydroxylase 1) involved in the biosynthesis of carnitine, which is essential for fatty acid supply in *β*-oxidation.

#### ARA induced modulation of the progeny (F_1_) liver transcriptome

DEGs (adjusted p<0.05) from F_1_ generation were sent to downstream analysis using IPA^®^ (S13 Table). Parental high ARA diet was associated with differential expression of a diverse set of genes clustering in several canonical pathways of which ‘eIF2 signalling pathway’ was predicted to be the most significantly downregulated pathway (Table 2).

**Table 2:** Significant canonical pathways associated with DEGs from F_1_ high ARA livers proposed by IPA^®^.

Canonical Pathways	p-value	z-score^1^	Gene symbols ^2^
**EIF2 Signaling**	3.98E-13	-2.887	RPL32,RPL11,RPL36A,RPS27,RPS8,RPS18,RPL29,EIF4G3,EIF2S1,XIAP,RPS28,RPS7,RPS26,SREBF1,RPL19,RPL21,RPL5,RPS25,RPS15A,RPS2,RPL36,RPS17,RPL18,RPL38
**LXR/RXR Activation**	5.13E-03	-2.000	APOB,C3,SREBF1,FASN,ACACA,SERPINA1,RXRA
**Neuropathic Pain Signaling In Dorsal Horn Neurons**	4.47E-02	-1.342	PLCD3,PLCE1,PRKCQ,PRKAG2,GRM6
**Melatonin Signaling**	3.80E-02	-1.000	PLCD3,PLCE1,PRKCQ,PRKAG2
**PPARα/RXRα Activation**	3.31E-03	0.447	PLCD3,PLCE1,GPD1,HELZ2,ACOX1,FASN,PRKAG2,RXRA,ADIPOR1
**LPS/IL-1 Mediated Inhibition of RXR Function**	3.89E-04	0.447	ALDH1L1,SCARB1,CYP3A4,SREBF1,ACOX1,SULT1A1,ALDH1A2,SULT1A3/SULT1A4,FABP7,GSTA1,RXRA,FMO5
**Sperm Motility**	2.82E-02	-0.447	PLCD3,PLCE1,PRKCQ,CACNA1H,PRKAG2,PDE4B
**Regulation of eIF4 and p70S6K Signaling**	2.63E-06		ITGB1,RPS27,RPS8,RPS18,EIF4G3,EIF2S1,RPS28,RPS7,RPS26,RPS25,RPS15A,RPS2,RPS17
**mTOR Signaling**	7.76E-06		RPS28,RPS7,DGKZ,PRKCQ,RPS26,RPS27,RPS18,RPS8,PRKAG2,EIF4G3,RPS25,RPS15A,RPS2,RPS17
**Polyamine Regulation in Colon Cancer**	3.02E-05		AZIN1,SAT2,PSME4,OAZ1,OAZ2
**Unfolded protein response**	2.45E-04		HSPA4,SREBF1,EDEM1,HSPA9,VCP,CANX
**Dopamine Degradation**	1.20E-03		ALDH1L1,COMT,SULT1A1,ALDH1A2,SULT1A3/SULT1A4
**Cysteine Biosynthesis III (mammalia)**	1.78E-03		CBS/CBSL,MAT1A,CTH,PRMT1
**FXR/RXR Activation**	1.91E-03		APOB,C3,SCARB1,SREBF1,FASN,SERPINA1,RXRA,VLDLR
**Aryl Hydrocarbon Receptor Signaling**	2.82E-03		ALDH1L1,TFDP1,ALDH1A2,GSTA1,RXRA,ESR1,PTGES3,AHR
**Protein Ubiquitination Pathway**	3.80E-03		B2M,HSPA4,UBE2D2,UBE4B,UBE2H,DNAJB11,DNAJC19,HSPA9,HSPD1,THOP1,XIAP
**Superpathway of Methionine Degradation**	4.47E-03		CBS/CBSL,MAT1A,GOT1,CTH,PRMT1
**Histidine Degradation VI**	5.37E-03		CYP46A1,UROC1,MICAL2
**Caveolar-mediated Endocytosis Signaling**	6.92E-03		ITGB1,B2M,COPG2,COPB2,COPB1
**Cysteine Biosynthesis/Homocysteine Degradation**	7.08E-03		CBS/CBSL,CTH
**Citrulline Biosynthesis**	8.51E-03		LOC102724788/PRODH,OAT,GLS2
**Xenobiotic Metabolism Signaling**	8.51E-03		ALDH1L1,PRKCQ,CYP3A4,SULT1A1,ALDH1A2,SULT1A3/SULT1A4,GSTA1,RXRA,FMO5,PTGES3,AHR
**Aldosterone Signaling in Epithelial Cells**	8.71E-03		PLCD3,HSPA4,PLCE1,PRKCQ,DNAJB11,DNAJC19,HSPA9,HSPD1
**tRNA Charging**	1.12E-02		LARS,CARS,TARS,VARS,QARS
**GPCR-Mediated Nutrient Sensing in Enteroendocrine Cells**	1.78E-02		PLCD3,PLCE1,PRKCQ,PRKAG2,TAS1R3
**Putrescine Degradation III**	1.95E-02		ALDH1L1,ALDH1A2,SAT2
**Superpathway of Citrulline Metabolism**	2.40E-02		LOC102724788/PRODH,OAT,GLS2
**Prostanoid Biosynthesis**	2.45E-02		PTGDS,PTGES3
**PXR/RXR Activation**	2.75E-02		CYP3A4,PRKAG2,GSTA1,RXRA
**TR/RXR Activation**	2.95E-02		SCARB1,SREBF1,FASN,ACACA,RXRA
**Pregnenolone Biosynthesis**	3.09E-02		CYP46A1,MICAL2
**Neuroprotective Role of THOP1 in Alzheimer's Disease**	3.89E-02		PRKAG2,THOP1,ACE
**VDR/RXR Activation**	4.07E-02		SERPINB1,YY1,PRKCQ,RXRA
**RAR Activation**	4.37E-02		PRKCQ,ALDH1A2,PRKAG2,RBP2,SMARCD1,RXRA,PRMT1
**Endoplasmic Reticulum Stress Pathway**	4.57E-02		EIF2S1,TAOK3
**Glucose and Glucose-1-phosphate Degradation**	5.01E-02		RGN,PGM2
**Pentose Phosphate Pathway**	5.01E-02		PGLS,RPIA
**Phagosome Formation**	5.01E-02		ITGB1,MRC1,PLCD3,PLCE1,PRKCQ

^1^ IPA^®^ predicts upregulation (positive z-score) or downregulation (negative z-score) of canonical pathways.

^2^ Gene symbols are reported as human orthologue gene symbols.

**Fig 4:**
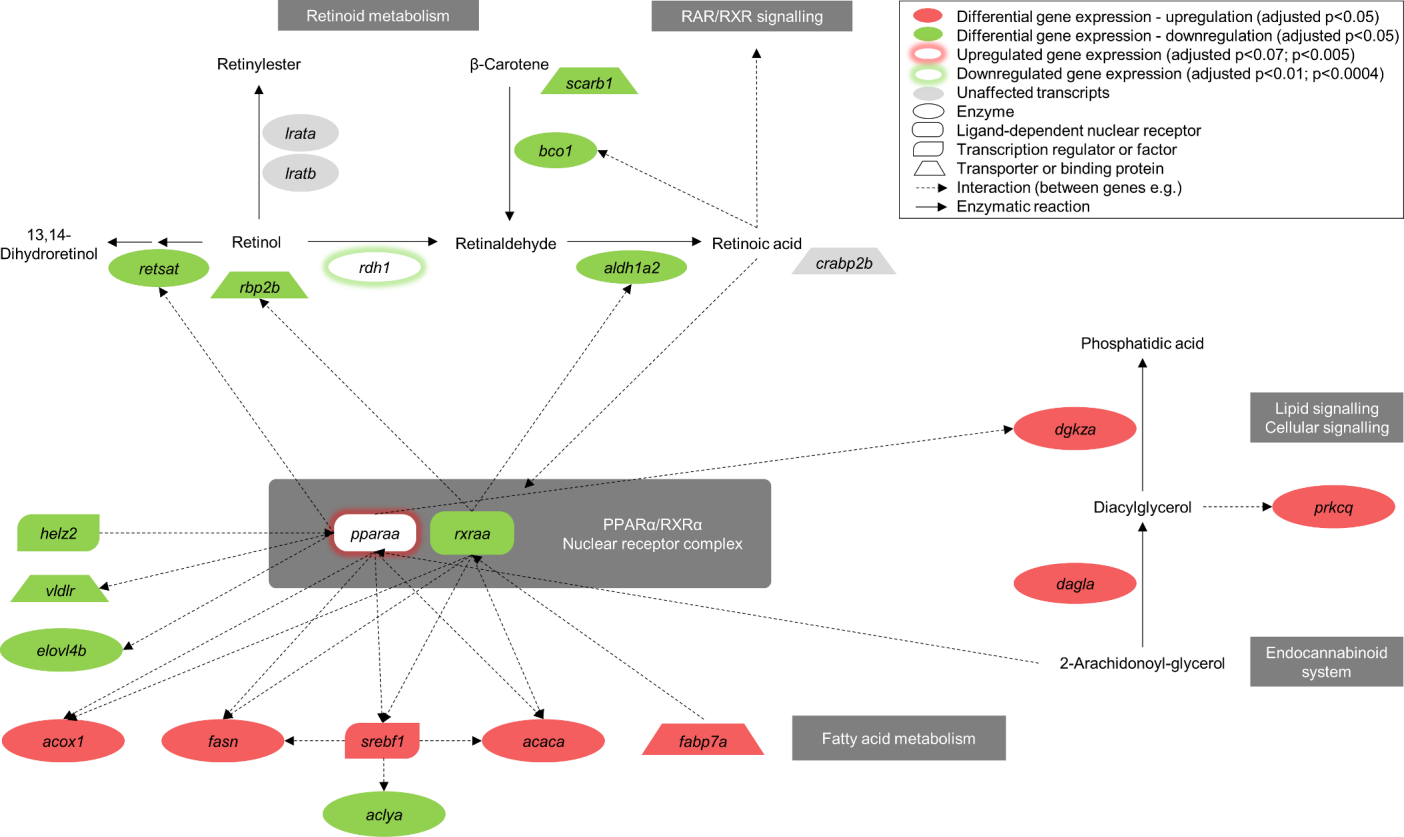
Differential expression of genes involved in retinoid processing and putative interactions with the PPARα/RXRα pathway and lipid signalling in male livers that are associated with parental high dietary ARA levels. Filled shapes in the figure represent overlapping differentially expressed genes between RefSeq and Ensembl annotation. Shapes that are highlighted with glow underlie gene expression information exclusively from Ensembl annotation (S4 Table); a*caca* (acetyl-CoA carboxylase alpha), *aclya* (ATP citrate lyase a); *acox1* (acyl-CoA oxidase 1, palmitoyl; alias: peroxisomal acyl-CoA oxidase 1); *aldh1a2* (aldehyde dehydrogenase 1 family, member A2); *bco1* (beta-carotene oxygenase 1); *crabp2b* (cellular retinoic acid binding protein 2, b); *dagla* (diacylglycerol lipase, alpha); *dgkza* (diacylglycerol kinase, zeta a), *elovl4b* (ELOVL fatty acid elongase 4b); *fabp7a* (fatty acid binding protein 7, brain, a); *fasn* (fatty acid synthase); *helz2* (helicase with zinc finger 2, transcriptional coactivator); *lrata* (lecithin retinol acyltransferase a (phosphatidylcholine-retinol O-acyltransferase)); *lratb* (lecithin retinol acyltransferase b (phosphatidylcholine-retinol O-acyltransferase)); *pparaa* (peroxisome proliferator-activated receptor alpha a); *prkcq* (protein kinase C, theta); *rbp2b* (retinol binding protein 2b, cellular); *rdh1* (retinol dehydrogenase 1); *retsat* (retinol saturase (all-*trans*-retinol 13,14-reductase)); *rxraa* (retinoid X receptor, alpha a); *scarb1* (scavenger receptor class B, member 1); *srebf1* (sterol regulatory element binding transcription factor 1); *vldlr* (very low density lipoprotein receptor).

Among the lipid metabolism related biological functions, the top most significantly enriched functions are shown in Table 3. The full list is given in S5 File where various biological functions related to phospholipid, steroid, long chain fatty acid and cholesterol metabolism were enriched by the F_1_ DEGs. ‘LXR/RXR activation’ canonical pathway is predicted to be downregulated (z = -2; Table 2). ‘PPARα/RXRα Activation’ was found to be a significantly enriched canonical pathway in the high ARA group. *Pparaa* showed higher expression levels (p = 0.005, S4 Table) as shown in Fig 4. ‘Oxidation of fatty acids’ is one of the top most enriched biological functions (Table 3). For *acox1* being involved in the first enzymatic step during peroxisomal *β*-oxidation and acting downstream of PPARα, showed upregulated expression in high ARA livers (Fig 4). *Helz2* which encodes a nuclear transcriptional co-activator for PPARα, was downregulated. High ARA livers showed an upregulated expression of genes involved in the long-chain fatty acid biosynthesis (*acaca*, *fasn* and *srebf1*) compared to livers arriving from control group (Fig 4). *Elovl4b*, which is involved in very long-chain fatty acid elongation was found to be significantly downregulated in high ARA livers. We found an upregulated gene expression for *dagla* that is involved in the synthesis of 2-arachidonoyl-glycerol, an endocannabinoid. Genes like *prkcq* and *dgkza* play roles in lipid signalling pathways like T cell receptor signalling and showed higher gene expression levels in high ARA group compared to control group.

**Table 3:** The top most significantly enriched lipid metabolism related biological functions associated with DEGs from F_1_ high ARA livers proposed by IPA^®^.

Biological Functions	p-value	z-score ^1^	Gene symbols ^2^
**Concentration of phospholipid**	3.82E-04	1.969	ACACA,CBS/CBSL,CHKA,DGKZ,FASN,LYST,NPC2,PITPNB,PLPP2,RGN,SCARB1,SREBF1,VLDLR
**Clearance of lipid**	5.72E-03	-1.960	C3,CYP3A4,SCARB1,VLDLR
**Oxidation of fatty acid**	2.04E-03	1.622	ACACA,ACOX1,ADIPOR1,C3,CYP3A4,FASN,PDK4,PRKAG2,SLC25A17,SLCO2A1,SREBF1
**Synthesis of lipid**	3.27E-06	-1.375	ACACA,ACLY,AHR,AKR1B1,ALDH1A2,APOB,ATP1A1,BCO1,C3,CACNA1H,CD9,CERS5,CHKA,CREB3L3,CYP39A1,CYP3A4,CYP46A1,DAGLA,DGKZ,ELOVL4,ESR1,FASN,FDX1,GSTA1,IGFBP2,ITGB1,LEPR,NPC2,PARK7,PDK4,PLCE1,PRKAG2,PTGDS,PTGES3,RGN,RXRA,SCARB1,SERPINA1,SH3KBP1,SREBF1
**Concentration of choline-phospholipid**	6.15E-03	1.342	ACACA,CHKA,FASN,LYST,SREBF1
**Transport of fatty acid**	4.49E-03	1.257	ABCC6,FABP7,SCARB1,SLC13A3,SLC25A17,SLCO2A1
**Concentration of acylglycerol**	2.26E-06	1.145	ACACA,ACLY,ADIPOR1,AKR1B1,APOB,ATP2A2,C3,CBS/CBSL,CHKA,CREB3L3,CYP3A4,DAGLA,FASN,FMO5,HELZ2,LEPR,MGLL,PDK4,RGN,RXRA,SCARB1,SREBF1,STEAP4,VLDLR
**Synthesis of terpenoid**	4.41E-04	-1.131	ACLY,AHR,ALDH1A2,APOB,ATP1A1,BCO1,CACNA1H,CYP39A1,CYP46A1,ESR1,FDX1,GSTA1,IGFBP2,PRKAG2,SCARB1,SERPINA1,SREBF1
**Incorporation of lipid**	6.20E-03	-1.127	ACLY,C3,FASN,SCARB1
**Concentration of progesterone**	5.80E-03	-1.067	CBS/CBSL,COMT,ESR1,LEPR,SCARB1

^1^ IPA^®^ predicts upregulation (positive z-score) or downregulation (negative z-score) of canonical pathways.

^2^ Gene symbols are reported as human orthologue gene symbols.

‘Metabolism of retinoid’ was among the enriched lipid metabolism related biological functions for F_1_ DEGs (S5 File). Transcripts of genes encoding enzymes involved in retinaldehyde synthesis from beta-carotene (*bco1*) and subsequent synthesis to retinoic acid (*aldh1a2*) as well as retinol saturation (*retsat*) were found to be downregulated in the high ARA group (Fig 4). *Rxraa* that is transcriptionally regulated by stereoisomers of retinoic acid, was shown to be downregulated in livers associated with parental high ARA diet. Two transcripts encoding transporters for beta-carotene (*scarb1*) and retinol (*rbp2b*) were downregulated.

**Fig 5:**
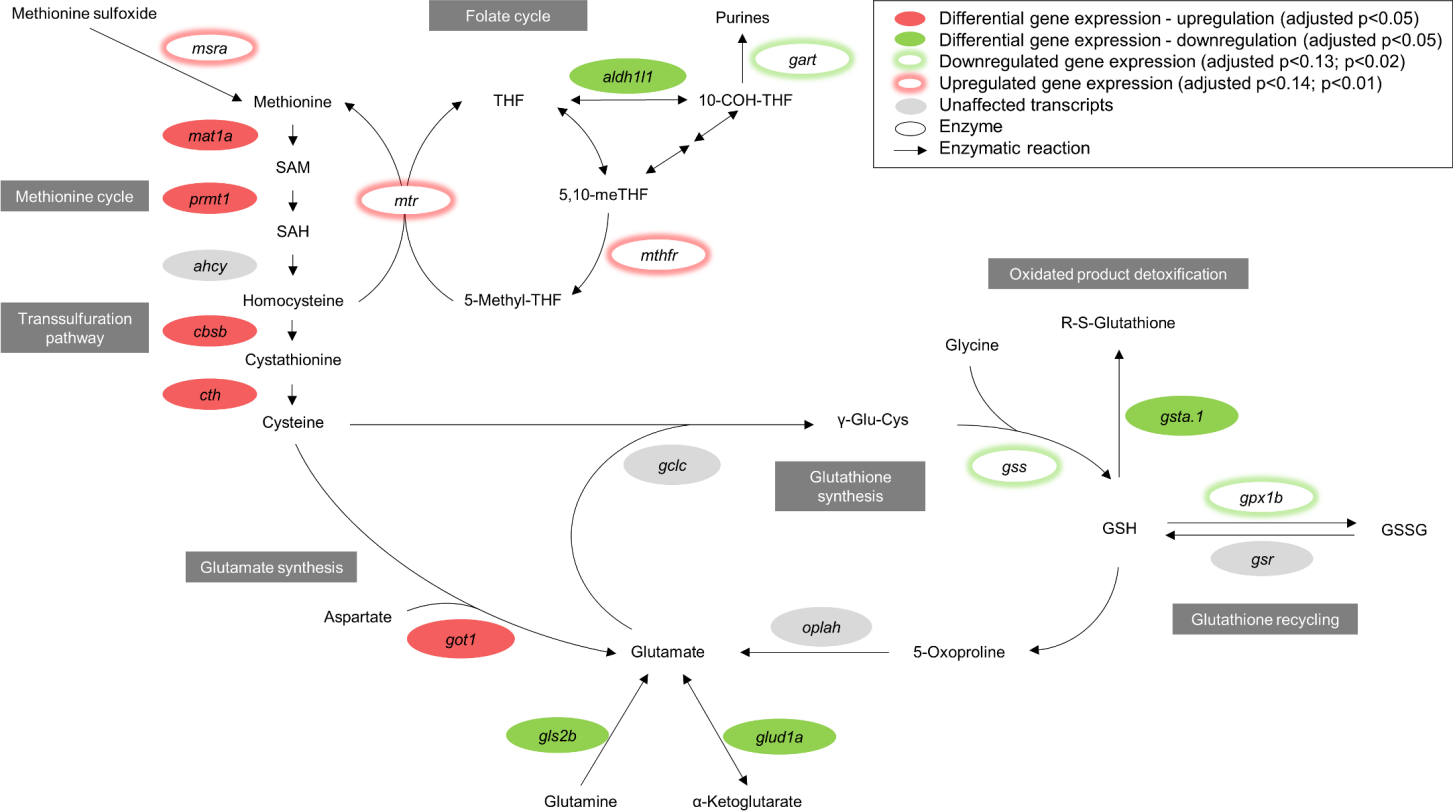
Parental high ARA levels are associated with differential expression of genes involved in methionine cycle, transsulfuration pathway, and glutamate and glutathione metabolism in male F_1_ livers. Filled shapes in the figure represent overlapping differentially expressed genes between RefSeq and Ensembl annotation. Shapes that are highlighted with glow underlie gene expression information exclusively from Ensembl annotation (S4 Table); *ahcy* (adenosylhomocysteinase); *aldh1l1* (aldehyde dehydrogenase 1 family, member L1); *cbsb* (cystathionine-beta-synthase b); *cth* (cystathionase (cystathionine gamma-lyase)); *gart* (phosphoribosylglycinamide formyltransferase); *gclc* (glutamate-cysteine ligase, catalytic subunit); *gls2b* (glutaminase 2b (liver, mitochondrial); *glud1a* (glutamate dehydrogenase 1a); *got1* (glutamic-oxaloacetic transaminase 1); *gpx1b* (glutathione peroxidase 1b); GSH (reduced glutathione); *gsr* (glutathione-disulfide reductase); *gss* (glutathione synthetase); GSSG (oxidized glutathione); *gsta*.*1* (glutathione S-transferase, alpha tandem duplicate 1); *mat1a* (methionine adenosyltransferase I alpha); *msra* (methionine sulfoxide reductase A); *mthfr* (methylenetetrahydrofolate reductase (NAD(P)H)); *mtr* (5-methyltetrahydrofolate-homocysteine methyltransferase); *oplah* (5-oxoprolinase (ATP-hydrolysing)); *prmt1* (protein arginine methyltransferase 1); R-S-glutathione (glutathione-S-conjugate); SAH (S-adenosyl-homocysteine); SAM (S-adenosyl-methionine); γ-Glu-Cys (gamma-glutamyl-cysteine); THF (tetrahydrofolate); 5-methyl-THF (5-methyltetrahydrofolate); 10-COH-THF (10-formyl-tetrahydrofolate); 5,10-meTHF (5,10-methylene-THF).

Among other enriched canonical pathways (Table 2), we found significantly upregulated transcripts of *mat1a*, *prmt1*, *cbsb*, *cth* and *got1* that clustered in ‘cysteine biosynthesis III (mammalian)’, ‘cysteine biosynthesis/homocysteine degradation’ and ‘superpathway of methionine degradation’ (Fig 5). Cysteine is provided through the transsulfuration pathway (*cbcb*, *cth*) for glutathione metabolism where *gsta*.*1* involved in glutathione detoxification was found to be downregulated in the high ARA group. *gls2b* and *glud1a* that are related to glutamate metabolism showed decreased expression in high ARA livers (Fig 5). *aldh1l1*, which is involved in the folate cycle, was downregulated in high ARA livers compared to control livers.

We observed increased gene expression of the estrogen receptor 1 (*esr1*) in livers associated with parental high ARA levels. Steroid metabolism related functions such as ‘concentration of progesteron’, ‘metabolism of estrogen’, ‘sulfation of beta-estradiol’ were suggested to be enriched by IPA^®^ (Table 3 and S5 File). Among the unmapped IDs, vitellogenin 5 (*vtg5*, no human orthologue) expression was found to be upregulated in high ARA livers compared to the control. In F_0_ high ARA livers, both *vtg5* (p = 0.01) and *esr1* (p = 0.02) showed decreased expression (S2 Table), but did not meet the chosen threshold requirements (adjusted p<0.05) for downstream analysis.

None of the F_1_ DEGs were in matching with those in F_0_ generation due to the chosen cut-off threshold.

#### Intergenerational differential gene expression

7.9% of the DEGs were overlapping between the intergenerational control and high ARA group comparison (S3 File). Functional annotation for KEGG pathways and GO terms of DEGs between both F_0_ and F_1_ control groups, and between F_0_ and F_1_ high ARA groups are given in S14 Table. Comparing the control groups, significantly enriched KEGG pathways and GO terms (q-value cut-off <0.05) related to amino acid biosynthesis and oxidoreduction related processes among others were identified. For the high ARA groups, mainly transcript processing, translation and protein biosynthesis related functions were identified.

#### Confirmatory RT-qPCR for RNA-sequencing validation

Expression of *fasn*, *vtg5*, *cbsb* and *mat1a* were investigated in the F_1_ livers by real-time RT-qPCR for RNA-seq validation purposes. RT-qPCR and RNA-seq derived expression patterns of those target genes were similar comparing means of control and high ARA replicates (S2 File).

## Discussion

In the present study we identified changes in hepatic gene expression patterns in the adult zebrafish progeny of parental fish given high dietary ARA levels. Despite the large mRNA expression differences observed in F_1_ generation, surprisingly few DEGs were found in F_0_ generation. The parental fish (F_0_) that were given the high ARA or control diet for 17 days (44 DPF) showed major metabolic profile differences as investigated in our previous article [21]. Thus it is difficult to explain the low effect at gene expression level in the parental generation. Variation in gene expression is often invoked to explain metabolic differences [64]. In addition, for this study we found no overlap between the two generations, and even reducing the cut-off stringency for DEGs in F_0_ generation gave very few genes overlapping between F_0_ and F_1_ generation. Furthermore, the differences between F_0_ control and F_1_ control patterns were larger than expected. One would expect that they cluster closer together as both were fed the control diet. Our data indicates that intergenerational differences in gene expression are greater than the intragenerational differences between the dietary groups. Although many factors might apply, it is conceivable that varying age of the fish as well as individual differences between the generations can give differences. Possibly, a differently composed diet fed to previous generations of the present F_0_ generation might have influenced F_0_ transcriptomic patterns. The latter one would also explain the small differential gene expression between F_0_ control and high ARA group. We can also not exclude the possibility of introducing bias due to different sampling points. Since it is difficult to identify the factors causing the intergenerational differences, we are obliged to be cautious interpreting the differences with regards to changes in pathways. Future studies should be conducted to reveal the significance of these results. The intergenerational analysis can be viewed in the supplementary files.

When comparing body weight in F_0_ and F_1_ generation, we previously reported a difference in F_0_ juveniles, whereas this effect disappeared at later stages (91 DPF) [21]. Similar, there were no differences in body weight between the two groups of adult F_1_ progeny.

Despite the weak coherence between transcriptomic and metabolic profile in F_0_, there was a link between the parental metabolic data and the gene expression in the F_1_ progeny. In the previous metabolomics study of the F_0_ parental fish, we observed decreased levels of glutathione, glutamine and cysteine and increased levels of oxidized metabolites of cysteine and methionine derivatives indicating an oxidized environment due to increased n-6 PUFA levels. Similarly in the present study, transcriptomic analysis of progeny livers (F_1_ high ARA) implied an increased expression of genes involved in the methionine cycle, cysteine (transsulfuration) and glutamate synthesis (Fig 5). This can in turn involve alterations in pathways such as glutathione or folate-mediated 1-C metabolism by affecting redox homeostasis or methylation-dependent functions such as for epigenetic modifications. The findings described above indicate major compensatory mechanisms in the progeny most likely owing a modulation of the transcriptome by the oxidized and pro-inflammatory environment previously reported in the parental fish fed high ARA. Despite giving both progeny groups the control diet, we found major differences in hepatic transcriptomic profiles at adult stage.

The present results suggest that parental high dietary ARA affected RXR activated pathways in the progeny. ‘PPARα/RXRα Activation’ and ‘LXR/RXR Activation’ pathways were among the top affected pathways according to the IPA^®^ analysis. Several genes belonging to retinol metabolic pathway were differentially expressed in adult progeny. Retinoids and their metabolites are potent activators controlling a range of essential physiological processes such as growth, limb patterning, eye vision, spermatogenesis and cell differentiation [65, 66]. Regulating their action is important for normal embryo development and epithelial differentiation, and disruption of signalling can have detrimental effects on the organism [67–70].

Retinoid metabolites act on lipid signalling pathways by activating RXRs which in turn form heterodimers with PPAR-lipid complexes (Fig 4). The PPAR complex controls transcription of target genes involved in lipid signalling and metabolism [71–73]. Several genes acting downstream of the nuclear receptor PPARα/RXRα complex were also found to be differently expressed in F_1_ high ARA livers. Genes, which encode enzymes regulating fatty acid levels, particularly by influencing fatty acid biosynthesis, transport and peroxisomal *β*-oxidation. Modulating these pathways influence energy expenditure, membrane composition and fatty acid distribution. In addition, oxidation of fatty acids were among the top lipid metabolic pathways enriched in the F_1_ high ARA group. We reported previously that elongated ARA metabolites and dicarboxylic acid levels were increased in the parental high ARA group [21]. Those results suggested an increased *β*-oxidation, particularly peroxisomal *β*-oxidative degradation of long-chain fatty acids, which can be associated with the observed transcriptomic changes related to fatty acid metabolism in the progeny.

Furthermore, studies have also shown a link between lipid and folate metabolism where PPARα seem to be involved in the regulation of key enzymes along the choline oxidative pathway which is closely linked to the methionine cycle [74, 75]. In the present study IPA^®^ reported the methionine degradation pathway as one of the significantly affected pathways in the progeny, despite no indicated direction of influence (z-score). It has been previously shown that the expression of genes regulating homocysteine synthesis from methionine were sensitive to either high dietary n-3 or n-6 PUFAs [76]. It is conceivable that our above described gene expression changes in fatty acid metabolism and the observed expression changes of genes involved in transsulfuration pathway and methionine cycle are linked.

Increased transcripts of estrogen receptor 1 and vitellogenin 5 were observed in male progeny livers associated with parental high dietary ARA levels. Interestingly, the same transcripts showed oppositely directed gene expression (regardless the chosen threshold) in the parental fish (F_0_ high ARA) suggesting compensatory mechanisms being involved. Vitellogenin, a lipid transporting protein, is produced in the liver and transported to the ovary as an egg yolk protein, under the regulation of estrogens in female fish [77–79]. Dietary ARA has been suggested to affect oocyte maturation and especially steroidogenesis in fish, but knowledge on mechanisms and impact on the following generations is lacking [80–82]. Induction of vitellogenin in male fish is commonly used as a marker for endocrine disruption [28] and studies on zebrafish have shown that vitellogenin synthesis can be induced in male fish when exposed to an estrogen (steroid hormone) named 17*β*-estradiol [83, 84]. In addition, vitellogenin has also an immunological role facilitating the defence against virus and bacteria [85, 86]. The exact biological role of vitellogenin in male fish is not clear. In humans, actions of estrogens are mediated by estrogen receptors and their role has been linked to metabolic inflammation [87]. Estrogens can regulate various energy metabolism pathways and disturbance by endocrine disruptors has been discussed in development of obesity [88]. However, our results from livers of male zebrafish progeny suggest that hepatic gene regulation related to steroidogenesis and estrogen signalling are sensitive to parental dietary high ARA intake.

We hypothesise that the observed DEGs in F_1_ high ARA livers compared to the control group, were associated with parental diet, but the exact modulatory mechanisms are not known. One mechanism whereby maternal diet can impact the progeny’s physiological status is the contribution to the nutrient reservoir in the developing oocyte [28, 89, 90]. Maternal transcripts present in the fertilized egg can steer gene expression in the developing embryo [31, 91, 92]. Epigenetic modifications of the genome in the gametes have also been identified as likely mechanisms through which environmental conditions, such as diet, can affect progeny transcriptomic patterns [26, 93]. Previous studies on zebrafish have shown that nutritional status of the parents such as vitamin B or vitamin E deficiency altered the transcriptome of their embryos [32, 33] and the livers of adult offspring [34]. Dietary PUFAs have been shown to affect membrane composition, cell signalling, gene expression and metabolism of the developing oocyte and thereby being able to influence the development of the next generation [7, 38, 94, 95].

Zebrafish has become a favoured research tool to investigate both molecular biological processes and the importance of nutrition in developmental, health and disease outcomes in vertebrates [46, 51, 96, 97]. Due to genetic, anatomical and physiological similarities to other vertebrates, zebrafish can be a useful model to evaluate the influence of dietary profiles on gene expression that can persist throughout life and across multiple generations as shown for different teleost species before [32, 33, 47, 52, 98]. Here, we demonstrated that parental diet affects the hepatic transcriptomic profiles in adult progeny. At the transcriptional level, we found that parental high ARA had a greater effect in the progeny than in the parents who were directly exposed to the experimental diets. These results are surprising, and show that the nutritional priming from parental generation has substantial effect on the progeny transcriptional profile. However, other factors could have influenced the lack of differential expression in the parental generation such as previous dietary treatments and time of sampling in relation to feeding. More knowledge is needed on how parental dietary habits can shape the progeny’s transcriptome and thereby possibly alter metabolic pathways in the progeny. Whether these differences can be inherited to further generations is an area for further research.

## Conclusions

Our work has shown that the parental diet modulated the transcription of a range of genes in the adult progeny connected to the fatty acid and retinoid metabolism, methionine cycle, transsulfuration pathway and estrogen signalling. We cannot distinguish if the effect at the transcriptional level is due to the nutritional composition of eggs (oocytes), maternal mRNA deposition or progeny transcriptome potential through programming of the gametes. Modulation of the transcriptome at early life stages can in turn affect metabolic pathways and their activity at later life stages. To our knowledge, the present study is the first one investigating liver transcriptome characteristics of adult zebrafish progeny from parents fed high ARA levels. Nevertheless, further study is required to understand deeper mechanisms on how those gene expression differences observed in adult progeny develop and if these effects can be transferred to future generations.

## Supporting information

S1 File Composition of control and high ARA diet. (PDF)

S2 File *Danio rerio* primer sequences used for real-time RT-qPCR of target genes for RNA-sequencing validation. (PDF)

S3 File Gene counts and overlap of significant differentially expressed genes (adjusted p<0.05) in F_0_ and F_1_ zebrafish livers obtained from RNA-sequencing and read mapping to the RefSeq and Ensembl reference genome (GRCz10). (PDF)

S4 File Volcano plot of RNA-sequencing data from male livers comparing control (A) and high ARA (B) group in F_0_ and F_1_ generation.
(PDF)

S5 File Ingenuity^®^ pathway analysis identified lipid metabolism associated biological functions represented by differentially expressed genes (adjusted p<0.05) from RNA-sequencing of F_1_ high ARA compared to control livers. (PDF)

S1 Table Differentially expressed genes in male F_0_ high ARA livers compared to control livers using the RefSeq genome. (CSV)

S2 Table Differentially expressed genes in male F_0_ high ARA livers compared to control livers using the Ensembl genome. (CSV)

S3 Table Differentially expressed genes in male F_1_ high ARA livers compared to control livers using the RefSeq genome. (CSV)

S4 Table Differentially expressed genes in male F_1_ high ARA livers compared to control livers using the Ensembl genome. (CSV)

S5 Table Differentially expressed genes in male F_0_ control livers compared to F_1_ control livers using the RefSeq genome. (CSV)

S6 Table Differentially expressed genes in male F_0_ control livers compared to F_1_ control livers using the Ensembl genome. (CSV)

S7 Table Differentially expressed genes in male F_0_ high ARA livers compared to F_1_ high ARA livers using the RefSeq genome. (CSV)

S8 Table Differentially expressed genes in male F_0_ high ARA livers compared to F_1_ high ARA livers using the Ensembl genome. (CSV)

S9 Table Concordant genes from the comparison of differentially expressed genes (adjusted p<0.05) between F_0_ control and F_0_ high ARA livers after mapping to the RefSeq and Ensembl reference genome (GRCz10). (CSV)

S10 Table Concordant genes from the comparison of differentially expressed genes (adjusted p<0.05) between F_1_ control and F_1_ high ARA livers after mapping to the RefSeq and Ensembl reference genome (GRCz10). (CSV)

S11 Table Concordant genes from the comparison of differentially expressed genes (adjusted p<0.05) between F_0_ and F_1_ control livers after mapping to the RefSeq and Ensembl reference genome (GRCz10). (CSV)

S12 Table Concordant genes from the comparison of differentially expressed genes (adjusted p<0.05) between F_0_ and F_1_ high ARA livers after mapping to the RefSeq and Ensembl reference genome (GRCz10). (CSV)

S13 Table Concordant genes in F_1_ generation uploaded into the Ingenuity^®^ Pathway Analysis software suite using human orthologues. (XLSX)

S14 Table Functional annotation of annotated differentially expressed genes between both F_0_ and F_1_ control and between F_0_ and F_1_ high ARA groups for KEGG pathways and GO terms. (XLSX)
