# Supplementary material for: Parental high dietary arachidonic acid levels modulated the hepatic transcriptome of adult zebrafish (Danio rerio) progeny
Source: PLoS One. 2018 Aug 2;13(8):e0201278. doi: 10.1371/journal.pone.0201278 (PMC6071982; doi:10.1371/journal.pone.0201278)
Supplement: S1 File — (PDF) [file pone.0201278.s001.pdf]

S1 File. Composition of control and high ARA diet.

Table 1. Ash, lipid, protein and energy composition.

	Ash (g/100g ww) [1]	Lipid (g/100g ww) [1]	Protein (g/100g ww) [1]	Energy (J/g ww) [1]
**Control diet**	6.8	12.2	50	21100
**High ARA diet**	7.0	12.9	50	21400

[1] Data are expressed as mean of two technical replicates.

Table 2. Ingredients and nutrient composition.

© Adam AC, Lie KK, Moren M, Skjaerven KH. *High dietary arachidonic acid levels induce changes in complex lipids and immune-related eicosanoids and increase levels of oxidised metabolites in zebrafish (Danio rerio).* Br J Nutr. 2017 May 09:1-11.

Ingredients	Control (g/kg DM)	High ARA (g/kg DM)
Protein blend *	767·9	767·9
Agar †	1·0	1·0
Fish oil ‡	8·0	8·0
Rape seed oil §	48·0	20·0
Flax seed oil §	20·0	4·0
Cargill´s ARA-rich oil ‖	4·0	48·0
Dextrin †	46·17	46·17
Cellulose ¶	19·3	19·3
Lecithin **	20·0	20·0
Mineral mix ††	50·0	50·0
Vitamin mix ‡‡	10·0	10·0
Methionine §§	2·5	2·5
Cyanocobalamin (1 %) ‖‖	0·99998	0·99998
Folic acid (97 %) ‖‖	0·0111	0·0111
Pyridoxine hydrochloride ‖‖	0·0199	0·0199
Astaxanthin ¶¶	0·3	0·3
Sucrose †	1·0	1·0
Tocopherol mix ***	0·75	0·75

ARA, arachidonic acid.

* BioMar AS products: fish meal, 5 %; krill meal, 1 %; soya protein concentrate, 6·2 %; maize, 5 %; wheat, 7·5 %; wheat gluten, 13 %; pea protein, 49·8 %; field peas, 12·5 %.

† Dissolved in 200 ml heated Milli-Q water, Sigma Aldrich Norway AS.

‡ Cod liver oil; Møllers, Axellus AS.

§ Rømer Produkt.

‖ Donated by Cargill (40 % ARA, Alking Bioengineering).

¶ Sigma Aldrich.

** Alfa Aesar.

†† Merck; ingredients (g/kg of diet): $CaHPO_4$ x $2H_2O$, 30; $CoCl_2$ x $6H_2O$, 0·007; $CuSO_4$ x $5H_2O$, 0·02; K_2SO_4, 15; KI, 0·05; $MgSO_4$ $7H_2O$, 5; $MnSO_4$ x H_2O, 0·05; NaCl, 2·873; Se-yeast, 0·2; $ZnSO_4$ x $7H_2O$, 0·5; $FeSO_4$ x $7H_2O$, 0·6.

‡‡ Obtained from Vilomix Norway AS, Norway; without cyanocobalamin, folic acid and pyridoxine hydrochloride (vitamin B_6) because of the trial set up with two directions (mg/kg of diet): vitamin A, 20; vitamin D, 4; vitamin E (50 %, acetate), 200; vitamin K (50 %), 10; vitamin C (35 %, phosphate), 350; choline, 1000; ascorbic acid, 1000; thiamine hydrochloride, 15; riboflavin (80 %), 19; nicotinamide, 200; inositol, 400; calcium pantothenate, 60; biotin (2 %), 50; filler (protein blend), 6672.

§§ Sigma-Aldrich.

‖‖ Normin AS.

¶¶ Dissolved in the agar solution; provided as a gift from G.O. Johnsen AS.

*** Provided as a gift from BASF.
