# Supplementary material for: Parental high dietary arachidonic acid levels modulated the hepatic transcriptome of adult zebrafish (Danio rerio) progeny
Source: PLoS One. 2018 Aug 2;13(8):e0201278. doi: 10.1371/journal.pone.0201278 (PMC6071982; doi:10.1371/journal.pone.0201278)
Supplement: S2 File — (PDF) [file pone.0201278.s002.pdf]

S2 File. *Danio rerio* primer sequences used for real-time RT-qPCR of target genes for RNA-sequencing validation.

Table 1. *Danio rerio* primer sequences used for real-time RT-qPCR.

Gene abbreviation	Gene name	Accession no.	Forward primer (5'-3')	Reverse primer (5'-3')	Amplicon size (bp)	PCR efficiency
**Reference genes:**						
tuba1c *	Tubulin, alpha 1c	NM_001105126.2	GGTGCCCTCAATGTGGATCT	GCCACAGAGAGCTGCTCATG	131	2.10
actb1	Actin, beta 1	NM_131031.1	TGCCCCTCGTGCTGTTTT	TCTGTCCCATGCCAACCAT	74	2.04
*eef1a1l1***	Eukaryotic translation elongation factor 1 alpha 1, like 1	NM_131263.1	AGACAACCCCAAGGCTCTCA	CTCATGTCACGCACAGCAAA	126	2.07
**Target genes:**						
fasn	Fatty acid synthase	XM_021472581.1	TGAACCCAGAGTCAGCACAC	ACGACAGTGCATTGTGAAAGG	116	2.4
vtg5	Vitellogenin 5	NM_001025189.2	CAGCTTGTTTTGGACAGGGC	GCAAGACTGGAGATGAGAGCC	101	2.2
mat1a	Methionine adenosyltransferase I, alpha	NM_199871.1	CAGGAGAACGGTGCTGTGAT	TCAGAATACGCTGCTGCTCC	100	2.15
cbsb	Cystathionine-beta-synthase b	NM_001014345.2	ACGGTGCTTCCCTTGGTTTC	CCATCCCCAGAATATGCCCC	106	2.12

* Olsvik PA, Williams TD, Tung HS, Mirbahai L, Sanden M, Skjaerven KH, et al. Impacts of TCDD and MeHg on DNA methylation in zebrafish (Danio rerio) across two generations. Comp Biochem Physiol C Toxicol Pharmacol. 2014 Sep;165:17-27.

** Skjærven KH, Jakt LM, Fernandes JMO, Dahl JA, Adam AC, Klughammer J, Bock C and Espe M. Parental micronutrient deficiency distorts liver DNA methylation and expression of lipid genes associated with a fatty-liver-like phenotype in offspring. Sci Rep. 2018. 8: 3055.

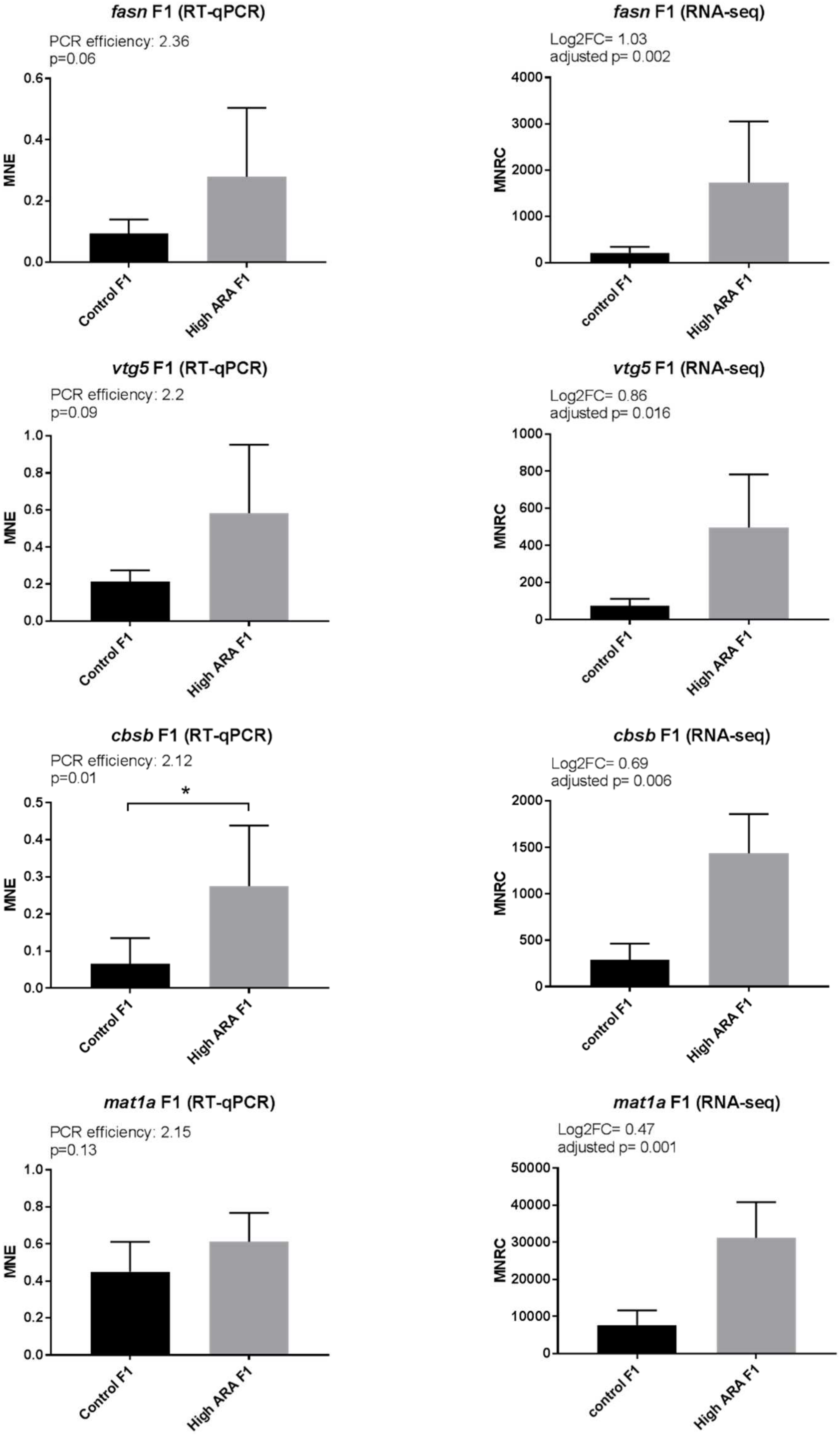

Figure 1. RNA-sequencing verification by real-time RT-qPCR. mRNA levels from RT-qPCR analysis are presented as mean normalized expression (MNE) and from RNA-sequencing as mean normalized read counts (MNRC, Ensembl) in F_1 high ARA versus F_1 control livers. mRNA levels of target genes were normalized against *tuba1c, actb1* and *eef1a1l1* as reference genes. Data presented are means ± SD of six replicates. Each replicate is a pool of six male livers from one feeding tank. p-values were calculated by a non-parametric *t*-test (Mann-Whitney test) using GraphPad Prism 6 software. Gene abbreviation, name and accession number are given in table 1 (S2_File).
