# Supplementary material for: Parental high dietary arachidonic acid levels modulated the hepatic transcriptome of adult zebrafish (Danio rerio) progeny
Source: PLoS One. 2018 Aug 2;13(8):e0201278. doi: 10.1371/journal.pone.0201278 (PMC6071982; doi:10.1371/journal.pone.0201278)
Supplement: S3 File — (PDF) [file pone.0201278.s003.pdf]

S3 File. Gene counts and overlap of significant differentially expressed genes (adjusted $p<0.05$) in F_0 and F_1 zebrafish livers obtained from RNA-sequencing and read mapping to the RefSeq and Ensembl reference genome (GRCz10).

Table 1. Gene counts of significant differentially expressed genes in F_0 and F_1 zebrafish livers obtained from RNA-sequencing.

	RefSeq			Ensembl			Concordant genes
	Up	Down	Sum	Up	Down	Sum	Sum
F_0 high ARA vs. F_0 control	27	12	39	19	8	27	20
F_1 high ARA vs. F_1 control	267	315	582	290	315	605	470
F_0 control vs. F_1 control	215	347	562	249	378	627	428
F_0 high ARA vs. F_1 high ARA	1180	1381	2561	1124	1283	2407	1987

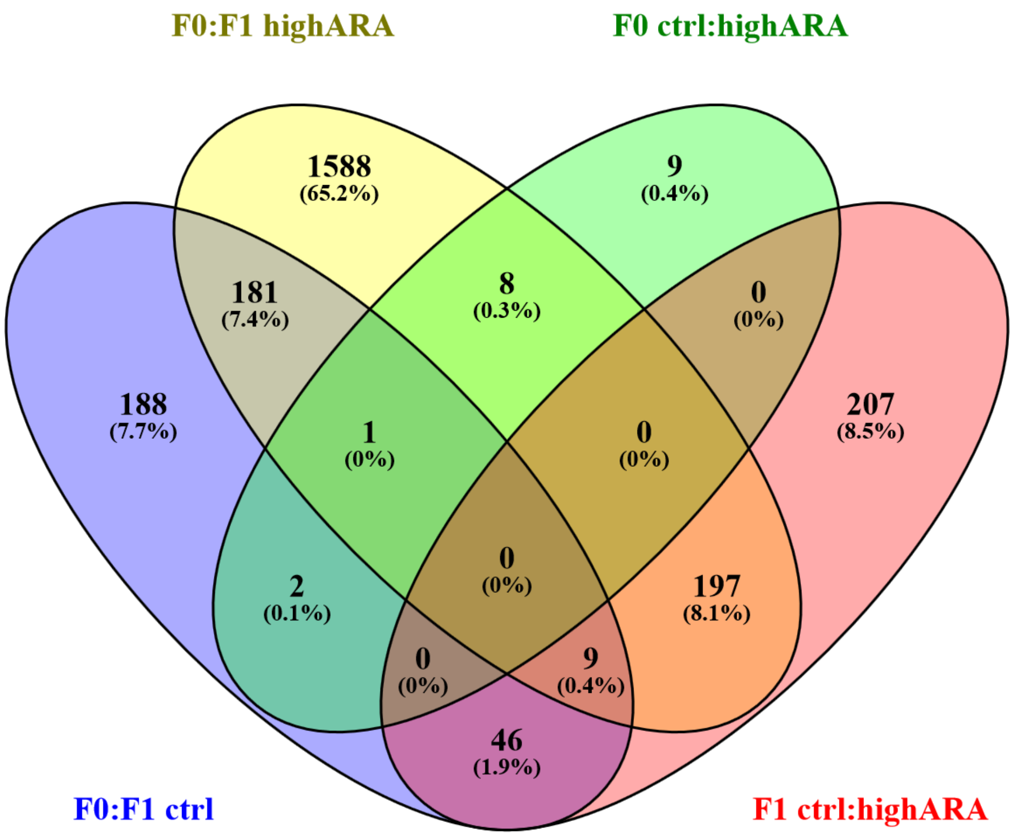

Figure 1. Venn diagram indicating overlap of gene symbols annotated to differentially expressed genes in F_0 and F_1 zebrafish livers obtained from RNA-sequencing.
