# Supplementary material for: Parental high dietary arachidonic acid levels modulated the hepatic transcriptome of adult zebrafish (Danio rerio) progeny
Source: PLoS One. 2018 Aug 2;13(8):e0201278. doi: 10.1371/journal.pone.0201278 (PMC6071982; doi:10.1371/journal.pone.0201278)
Supplement: S4 File — Volcano plot of RNA-sequencing data from male livers comparing control (A) and high ARA (B) group in F0 and F1 generation. (PDF) [file pone.0201278.s004.pdf]

S4 File. Volcano plot of RNA-sequencing data from male livers comparing control (A) and high ARA (B) group in F_0 and F_1 generation.

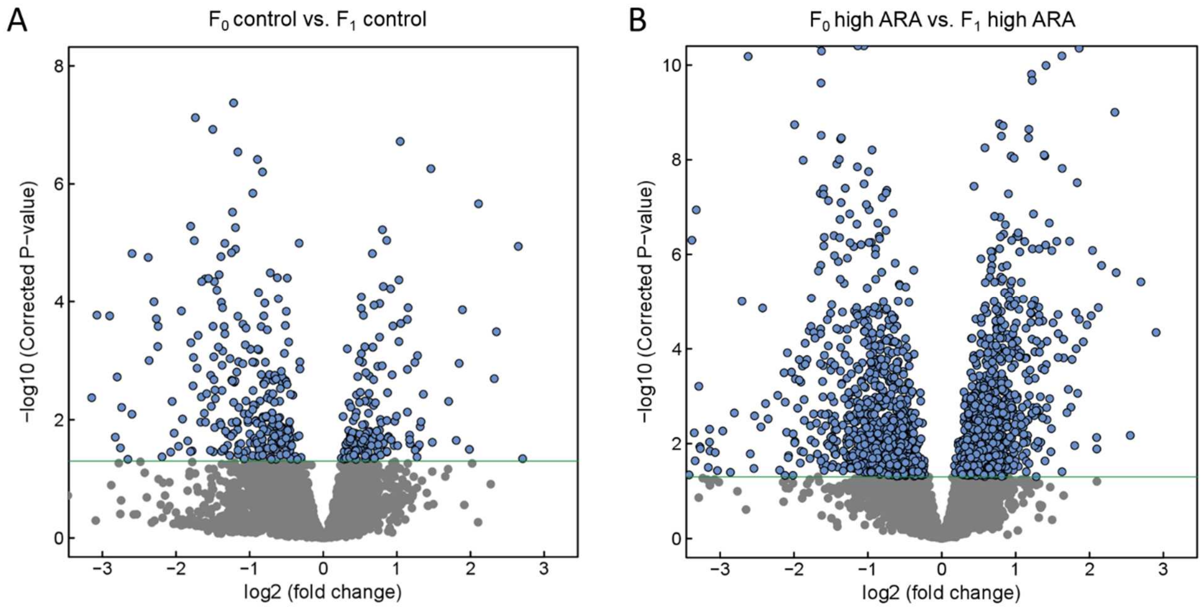

Figure 1. Volcano plot of RNA-sequencing data from male livers comparing control (A) and high ARA (B) group in F_0 and F_1 generation. Presented data represents overlapping genes from both RefSeq and Ensembl reference genome mapping (GRCz10). Blue spots represent DEGs of control (A) and high ARA (B) groups between F_0 and F_1 generation. The green line denoted the significance threshold (adjusted p<0.05) for DEGs.
