# Supplementary material for: Parental high dietary arachidonic acid levels modulated the hepatic transcriptome of adult zebrafish (Danio rerio) progeny
Source: PLoS One. 2018 Aug 2;13(8):e0201278. doi: 10.1371/journal.pone.0201278 (PMC6071982; doi:10.1371/journal.pone.0201278)
Supplement: S5 File — (PDF) [file pone.0201278.s005.pdf]

**S4 File. Ingenuity® Pathway Analysis identified lipid metabolism associated biological functions represented by differentially expressed genes (adjusted p<0.05) from RNA-sequencing of $F_1$ high ARA compared to control livers.**

| Biological Functions | p-value | z-score [1] | Genes [2] |
|---|---|---|---|
| **concentration of phospholipid** | 3.82E-04 | 1.969 | ACACA,CBS/CBSL,CHKA,DGKZ,FASN,LYST,NPC2,PITPNB,PLPP2,RGN,SCARB1,SREBF1,VLDLR |
| **clearance of lipid** | 5.72E-03 | -1.960 | C3,CYP3A4,SCARB1,VLDLR |
| **oxidation of fatty acid** | 2.04E-03 | 1.622 | ACACA,ACOX1,ADIPOR1,C3,CYP3A4,FASN,PDK4,PRKAG2,SLC25A17,SLCO2A1,SREBF1 |
| **synthesis of lipid** | 3.27E-06 | -1.375 | ACACA,ACLY,AHR,AKR1B1,ALDH1A2,APOB,ATP1A1,BCO1,C3,CACNA1H,CD9,CERS5,CHKA,CREB3L3,CYP39A1,CYP3A4,CYP46A1,DAGLA,DGKZ,ELOVL4,ESR1,FASN,FDX1,GSTA1,IGFBP2,ITGB1,LEPR,NPC2,PARK7,PDK4,PLCE1,PRKAG2,PTGDS,PTGES3,RGN,RXRA,SCARB1,SERPINA1,SH3KBP1,SREBF1 |
| **concentration of choline-phospholipid** | 6.15E-03 | 1.342 | ACACA,CHKA,FASN,LYST,SREBF1 |
| **transport of fatty acid** | 4.49E-03 | 1.257 | ABCC6,FABP7,SCARB1,SLC13A3,SLC25A17,SLCO2A1 |
| **concentration of acylglycerol** | 2.26E-06 | 1.145 | ACACA,ACLY,ADIPOR1,AKR1B1,APOB,ATP2A2,C3,CBS/CBSL,CHKA,CREB3L3,CYP3A4,DAGLA,FASN,FMO5,HELZ2,LEPR,MGLL,PDK4,RGN,RXRA,SCARB1,SREBF1,STEAP4,VLDLR |
| **synthesis of terpenoid** | 4.41E-04 | -1.131 | ACLY,AHR,ALDH1A2,APOB,ATP1A1,BCO1,CACNA1H,CYP39A1,CYP46A1,ESR1,FDX1,GSTA1,IGFBP2,PRKAG2,SCARB1,SERPINA1,SREBF1 |
| **incorporation of lipid** | 6.20E-03 | -1.127 | ACLY,C3,FASN,SCARB1 |
| **concentration of progesterone** | 5.80E-03 | -1.067 | CBS/CBSL,COMT,ESR1,LEPR,SCARB1 |
| **concentration of long chain fatty acid** | 1.57E-03 | 1.067 | ACLY,DAGLA,SCARB1,SREBF1 |
| **transport of steroid** | 2.60E-03 | -1.026 | ABCA12,APOB,CANX,CYP3A4,CYP46A1,NPC2,SCARB1,SLC25A1,SREBF1,VLDLR |
| **export of cholesterol** | 9.21E-04 | -0.998 | ABCA12,APOB,CANX,CYP46A1,NPC2,SCARB1,SLC25A1,SREBF1,VLDLR |
| **catabolism of lipid** | 2.61E-05 | -0.931 | AHR,APOB,COMT,CYP39A1,CYP3A4,CYP46A1,DAGLA,HEXA,MGLL,SCARB1,VLDLR |
| **metabolism of membrane lipid derivative** | 2.61E-04 | -0.919 | ACLY,APOB,CD9,CERS5,CHKA,CYP39A1,CYP46A1,DGKZ,FASN,FDX1,HEXA,ITGB1,LEPR,LYST,NSDHL,RXRA,SCARB1,SERPINA1,SH3KBP1,SREBF1,VLDLR |
| **accumulation of sphingolipid** | 1.72E-03 | -0.857 | ALDH1L1,CERS5,ELOVL4,HEXA,NPC2 |
| **conversion of fatty acid** | 2.72E-04 | 0.842 | ACACA,ACLY,ACOX1,CYP3A4,FASN,SREBF1 |
| **exposure of lipid** | 1.58E-03 | -0.811 | CD9,CYP3A4,ITGB1,LGALS2,PRKCQ |
| **exposure of phospholipid** | 9.63E-03 | -0.811 | CD9,ITGB1,LGALS2,PRKCQ |
| **efflux of cholesterol** | 3.22E-03 | -0.695 | ABCA12,APOB,CANX,NPC2,SCARB1,SLC25A1,SREBF1,VLDLR |
| **concentration of lipid** | 2.49E-06 | 0.672 | ACACA,ACLY,ADIPOR1,AHR,AKR1B1,APOB,ATP1A1,ATP2A2,BCO1,C3,CBS/CBSL,CFD,CHKA,COMT,CREB3L3,CYP3A4,DAGLA,DGKZ,ESR1,FASN,FMO5,GNE,GSTA1,HELZ2,HMGN1,LEPR,LOC102724788/PRODH,LYST,MGLL,MRC1,NPC2,PDK4,PITPNB,PLPP2,PTGDS,PTGES3,RBP2,RGN,RXRA,SCARB1,SLCO2A1,SREBF1,STEAP4,VLDLR |
| **metabolism of terpenoid** | 1.33E-08 | -0.625 | ACLY,AHR,ALDH1A2,APOB,BCO1,CACNA1H,COMT,CYP39A1,CYP3A4,CYP46A1,ESR1,FDX1,GSTA1,LEPR,NPC2,NSDHL,PLEKHA1,RBP2,RETSAT,RXRA,SCARB1,SERPINA1,SREBF1,SULT1A1,VLDLR |
| **fatty acid metabolism** | 4.13E-06 | -0.601 | ABCA12,ABCC6,ACACA,ACLY,ACOX1,AKR1B1,APOB,CANX,CD9,CERS5,CHKA,CYP3A4,CYP46A1,ELOVL4,FABP7,FASN,FDX1,HBP1,LEPR,MGLL,NPC2,PARK7,PDK4,PRKAG2,PTGDS,PTGES3,RGN,RXRA,SCARB1,SLC13A3,SLC25A1,SLC25A17,SLCO2A1,SREBF1,VLDLR |
| **steroid metabolism** | 3.81E-06 | -0.581 | ACLY,APOB,CACNA1H,COMT,CYP39A1,CYP3A4,CYP46A1,ESR1,FDX1,GSTA1,LEPR,NSDHL,PLEKHA1,RXRA,SCARB1,SERPINA1,SREBF1,SULT1A1,VLDLR |
| **synthesis of steroid** | 1.24E-03 | -0.542 | ACLY,AHR,APOB,ATP1A1,CACNA1H,CYP39A1,CYP46A1,ESR1,FDX1,GSTA1,IGFBP2,PRKAG2,SCARB1,SERPINA1,SREBF1 |
| **accumulation of acylglycerol** | 4.35E-04 | 0.501 | ACACA,ADIPOR1,APOB,MGLL,PDK4,SCARB1,SREBF1,TAS1R3,VLDLR |
| **oxidation of lipid** | 2.58E-03 | 0.496 | ACACA,ACOX1,ADIPOR1,ALDH1A2,C3,CYP3A4,FASN,PDK4,PRKAG2,SCARB1,SLC25A17,SLCO2A1,SREBF1 |
| **concentration of sterol** | 1.10E-03 | 0.409 | AHR,APOB,ATP1A1,CBS/CBSL,CHKA,CYP3A4,ESR1,FMO5,LEPR,MGLL,NPC2,RGN,RXRA,SCARB1,SREBF1,STEAP4,VLDLR |
| **synthesis of acylglycerol** | 1.83E-03 | 0.264 | C3,CREB3L3,DAGLA,FASN,PLCE1,RGN,SCARB1,SREBF1 |
| **synthesis of fatty acid** | 6.58E-03 | -0.258 | ACACA,ACLY,AKR1B1,APOB,CYP3A4,ELOVL4,FASN,LEPR,NPC2,PARK7,PDK4,PTGDS,PTGES3,RGN,RXRA,SREBF1 |
| **accumulation of lipid** | 2.92E-06 | -0.257 | ACACA,ACOX1,ADIPOR1,AHR,ALDH1L1,APOB,BCO1,CERS5,COL14A1,ELOVL4,FABP7,FASN,HEXA,LEPR,MGLL,NPC2,PDK4,RETSAT,SCARB1,SREBF1,TAS1R3,VLDLR,YY1 |
| **concentration of cholesterol** | 1.45E-03 | 0.255 | AHR,APOB,ATP1A1,CBS/CBSL,CYP3A4,ESR1,FMO5,LEPR,MGLL,NPC2,RGN,RXRA,SCARB1,SREBF1,STEAP4,VLDLR |
| **metabolism of acylglycerol** | 2.15E-04 | -0.237 | APOB,C3,CREB3L3,DAGLA,DGKZ,FASN,MGLL,PLCE1,RGN,SCARB1,SREBF1 |
| **concentration of triacylglycerol** | 5.21E-06 | 0.228 | ACACA,ACLY,ADIPOR1,AKR1B1,APOB,ATP2A2,C3,CBS/CBSL,CHKA,CYP3A4,FASN,FMO5,HELZ2,LEPR,MGLL,PDK4,RGN,RXRA,SCARB1,SREBF1,STEAP4,VLDLR |

| | | | |
|---|---|---|---|
| **metabolism of cholesterol** | 1.04E-06 | -0.226 | ACLY,APOB,CYP39A1,CYP46A1,FDX1,LEPR,NSDHL,RXRA,SCARB1,SERPINA1,SREBF1,VLDLR |
| **concentration of fatty acid** | 1.91E-04 | 0.137 | ACACA,ACLY,AKR1B1,APOB,C3,CBS/CBSL,DAGLA,LEPR,LOC102724788/PRODH,MGLL,PDK4,PTGDS,PTGES3,RXRA,SCARB1,SLCO2A1,SREBF1,VLDLR |
| **transport of lipid** | 4.70E-05 | -0.099 | ABCA12,ABCC6,APOB,CANX,CHKA,CYP3A4,CYP46A1,FABP7,HBP1,NPC2,SCARB1,SLC13A3,SLC25A1,SLC25A17,SLCO2A1,SREBF1,VLDLR |
| **accumulation of triacylglycerol** | 1.30E-03 | 0.056 | ACACA,ADIPOR1,APOB,PDK4,SCARB1,SREBF1,TAS1R3,VLDLR |
| **flux of lipid** | 2.44E-03 | 0.005 | ABCA12,APOB,CANX,NPC2,SCARB1,SLC25A1,SLCO2A1,SREBF1,VLDLR |
| **catabolism of terpenoid** | 2.91E-06 | | AHR,COMT,CYP39A1,CYP3A4,CYP46A1,SCARB1,VLDLR |
| **catabolism of steroid** | 1.17E-05 | | COMT,CYP39A1,CYP3A4,CYP46A1,SCARB1,VLDLR |
| **catabolism of cholesterol** | 4.49E-05 | | CYP39A1,CYP46A1,SCARB1,VLDLR |
| **conversion of malonyl-coenzyme A** | 2.67E-04 | | ACACA,FASN |
| **metabolism of acetyl-coenzyme A** | 3.39E-04 | | ACACA,ACLY,FASN |
| **metabolism of retinoid** | 6.76E-04 | | AHR,ALDH1A2,BCO1,CYP3A4,RBP2,RETSAT |
| **synthesis of acyl-coenzyme A** | 6.80E-04 | | ACACA,ACLY,FASN,PDK4 |
| **synthesis of myristic acid** | 7.93E-04 | | FASN,SREBF1 |
| **concentration of malonyl-coenzyme A** | 7.93E-04 | | ACACA,ACLY,PDK4,SREBF1 |
| **distribution of cholesterol** | 8.57E-04 | | ATP1A1,CBS/CBSL,SCARB1 |
| **metabolism of estrogen** | 9.20E-04 | | COMT,CYP3A4,PLEKHA1,SULT1A1 |
| **conversion of cholesterol** | 1.10E-03 | | CYP3A4,CYP46A1,RXRA |
| **homeostasis of lipid** | 1.27E-03 | | ABCA12,ACACA,APOB,C3,CYP3A4,GOT1,LYST,MAT1A,NPC2,SCARB1,SREBF1 |
| **conversion of acyl-coenzyme A** | 1.38E-03 | | ACACA,ACLY,FASN |
| **synthesis of acetyl-coenzyme A** | 1.38E-03 | | ACLY,FASN,PDK4 |
| **synthesis of malonyl-coenzyme A** | 1.57E-03 | | ACACA,FASN |
| **abnormal quantity of lipid** | 1.85E-03 | | ESR1,HELZ2,MRC1,NPC2,RBP2,RGN,SCARB1,SLCO2A1,STEAP4 |
| **accumulation of asialo GM2 ganglioside** | 2.59E-03 | | HEXA,NPC2 |
| **sulfation of 2-hydroxyestradiol** | 2.59E-03 | | SULT1A1,SULT1A3/SULT1A4 |
| **synthesis of palmitic acid** | 3.47E-03 | | ACACA,FASN,SREBF1 |
| **mobilization of acylglycerol** | 3.84E-03 | | APOB,DAGLA |
| **sulfation of beta-estradiol** | 3.84E-03 | | SULT1A1,SULT1A3/SULT1A4 |
| **uptake of phospholipid** | 3.84E-03 | | PEBP1,SCARB1 |
| **catabolism of acylglycerol** | 4.03E-03 | | APOB,DAGLA,MGLL |
| **quantity of monounsaturated fatty acids** | 4.65E-03 | | ACLY,SCARB1,SREBF1 |
| **synthesis of sterol** | 5.23E-03 | | ACLY,APOB,CYP46A1,PRKAG2,SERPINA1,SREBF1 |
| **concentration of 1,2-dipalmitoylphosphatidylcholine** | 5.31E-03 | | ACACA,FASN |
| **conversion of acetyl-coenzyme A** | 5.31E-03 | | ACACA,ACLY |
| **quantity of non-esterified fatty acid** | 6.82E-03 | | AKR1B1,CBS/CBSL,MGLL |
| **accumulation of D-erythro-C16-ceramide** | 7.01E-03 | | ALDH1L1,CERS5 |
| **incorporation of glycosphingolipid** | 7.01E-03 | | ACLY,SCARB1 |
| **synthesis of cholesterol** | 7.74E-03 | | ACLY,APOB,CYP46A1,SERPINA1,SREBF1 |
| **abnormal quantity of cholesterol** | 8.55E-03 | | ESR1,SCARB1,STEAP4 |
| **secretion of cholesterol** | 8.55E-03 | | APOB,ESR1,SCARB1 |

[1] IPA® predicts upregulation (positive z-score) or downregulation (negative z-score) of biological functions.

[2] Gene names are reported as human orthologue gene symbols.
